# Plant Neurobiology, a Fascinating Perspective in the Field of Research on Plant Secondary Metabolites

**DOI:** 10.3390/ijms140610819

**Published:** 2013-05-23

**Authors:** Marcello Iriti

**Affiliations:** Department of Agricultural and Environmental Sciences, Milan State University, via G. Celoria 2, 20133 Milan, Italy; E-Mail: marcello.iriti@unimi.it

**Keywords:** indoleamines, melatonin, serotonin, catecholamines, dopamine, norepinephrine, epinephrine

## Abstract

In this Editorial, I comment on the exciting and original topic of plant neurobiology, focusing on natural products whose biosynthesis is shared by animal and plant organisms, *i.e.*, indoleamines (melatonin and serotonin) and catecholamines (dopamine, norepinephrine and epinephrine).

Some years ago, in a review article published in the *International Journal of Molecular Sciences*, I emphasized the phytochemical diversity as a main plant adaptive trait in a changing environment [1]. By means of secondary metabolites, plant organisms defend themselves from pathogen infections, phytophagy attacks and competition with other plants (allelopathy), as well as tolerating detrimental climatic conditions, environmental pollutants, high solar irradiance and water deficit. However, the most important ecological role of plant secondary metabolism is related to reproductive function, rather than defence, which guarantees the evolutionary success of any living species. Secondary metabolites include pigments and volatile compounds, conferring colours and scents, respectively, to flower organs and fruits, thus attracting pollinators and fruit predators and favouring fecundation and seed dispersion.

Despite recent advances in the field of research on plant secondary metabolism, in the metabolomic era only a few phytochemicals have been discovered and studied among the hundreds or thousands of secondary metabolites produced by plants, and, in some cases, the functional roles of some compounds in plants have not yet been completely clarified. In addition, some “original” molecules have been discovered in plants. This is the case of typical, but not exclusive, “animal” metabolites, such as the mammalian neurotransmitters and neurohormones indoleamines (melatonin and serotonin) and catecholamines (dopamine, norepinephrine and epinephrine) (Figure 1).

Melatonin (*N*-acetyl-5-methoxytryptamine) was first reported in food plants and medicinal herbs about two decades ago [2,3], and, more recently, it has been proposed as a healthy component of the traditional Mediterranean diet [4]. Because of its structural similarity with the plant growth hormones of the auxin family, a hormone-like role has been attributed to melatonin in some plant species, as well as an action in delaying flowering, preventing chlorophyll degradation, protecting against oxidative damage, abiotic stresses, pathogens and environmental pollutants [5]. Serotonin (5-hydroxytryptamine) has also been detected in many edible plants [6], where it has been implicated as having important physiological and developmental functions including flowering, senescence, shoot formation and defence responses [7]. Many plant species present in the human diet contain catecholamines, in the order: dopamine (3-hydroxytyramine) > norepinephrine (or noradrenaline) > epinephrine (or adrenaline) [8]. They are involved in some relevant regulatory functions in plant physiology, in concert with some phytohormones, such as growth, carbohydrate metabolism and some stress responses [8].

In conclusion to my opinion, a complete dichotomy between plant and animal metabolites may represent a misinterpretation. Plants are more ancient than animals, in evolutionary terms. They appeared earlier than animals on the earth and, presumably, they begun to synthesise catecholamines and indoleamines earlier than animals. In any case, plants and animals are not in the same evolutionary lineage, and, therefore, the biosynthesis of these molecules in so much different organisms may be due to a phenomenon of parallel evolution. Possibly, epinephrine and melatonin possess molecular moieties in their chemical structures, able to interact with conserved receptors or responsible for their antioxidant or other biological activities. I anticipate that, in the near future, an ever increasing number of researchers will be involved in this fascinating field of plant biology.

## Figures and Tables

**Figure 1:**
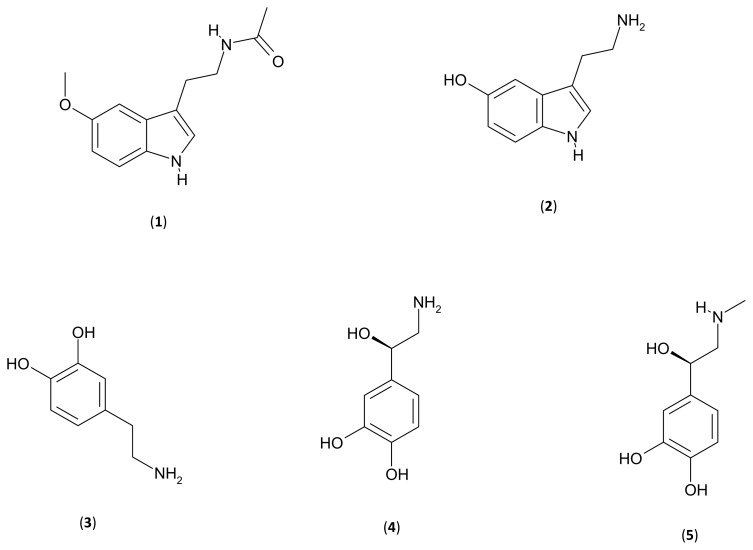
Chemical structures of the indoleamines (**1**) melatonin (*N*-acetyl-5-methoxytryptamine) and (**2**) serotonin (5-hydroxytryptamine), and the catecholamines (**3**) dopamine (3-hydroxytyramine), (**4**) norepinephrine (or noradrenaline) and (**5**) epinephrine (or adrenaline).
